# Effect of educational intervention on medication reconciliation practice of hospital pharmacists in a developing country – A non-randomised controlled trial

**DOI:** 10.1186/s12909-023-04844-7

**Published:** 2023-11-15

**Authors:** Akinniyi A. Aje, Segun J. Showande, Rasaq Adisa, Titilayo O. Fakeye, Oluwakemi A. Olutayo, Lawrence A. Adebusoye, Olufemi O. Olowookere

**Affiliations:** 1 Department of Clinical Pharmacy and Pharmacy Administration, Faculty of Pharmacy, University of Ibadan, Ibadan, Nigeria; 2 Pharmacy Department, Chief Tony Anenih Geriatric Centre, University College Hospital, Ibadan, Nigeria; 3 Chief Tony Anenih Geriatric Centre, University College Hospital, Ibadan, Nigeria

**Keywords:** Diabetes, Drug therapy problems, Educational intervention, Hospital pharmacist, Hypertension, Medication discrepancy, Medication reconciliation

## Abstract

**Background:**

Medication reconciliation is an evidence-based practice that reduces medication-related harm to patients. This study evaluated the effect of educational intervention on medication reconciliation practice of pharmacists among ambulatory diabetes and hypertensive patients.

**Methods:**

A non-randomized clinical trial on medication reconciliation practice was carried out among 85 and 61 pharmacists at the intervention site and control site, respectively. Medication reconciliation was carried out among 334 (intervention-183; control-151) diabetes and/or hypertensive patients by the principal investigator to indirectly evaluate pharmacists’ baseline medication reconciliation practice at both sites. A general educational intervention was carried out among intervention pharmacists. Medication reconciliation was carried out by the principal investigator among another cohort of 96 (intervention-46; control-50) and 90 (intervention-44; control-46) patients at three and six months postintervention, respectively, to indirectly assess pharmacists’ postintervention medication reconciliation practice. Thereafter, a focused educational intervention was carried out among 15 of the intervention pharmacists. Three experts in clinical pharmacy analysed the medication reconciliation form filled by the 15 pharmacists after carrying out medication reconciliation on another cohort of 140 patients, after the focused intervention. Data was summarized with descriptive (frequency, percentage, mean ± standard deviation) and inferential (Pearson product-moment correlations analysis, independent-samples t-test and one-way ANOVA) statistics with level of significance set at *p*<0.05.

**Key findings:**

Baseline medication reconciliation practice was poor at both sites. Post-general educational intervention, medication discrepancy was significantly reduced by 42.8% at the intervention site (*p*<0.001). At the intervention site, a significant increase of 54.3% was observed in patients bringing their medication packs for clinic appointments making medication reconciliation easier (*p*=0.003), at 6-months postintervention. Thirty-five, 66 and 48 drug therapy problems were detected by 31 (43.1%), 33 (66.0%) and 32 (71.1%) intervention pharmacists at 1-, 3- and 6-month post-general educational intervention, respectively. Post-focused educational intervention, out of a total of 695 medications prescribed, 75 (10.8%) medication discrepancies were detected and resolved among 42 (30%) patients by the 15 pharmacists.

**Conclusions:**

The educational interventions improved pharmacists’ medication reconciliation practice at the intervention site. It is expected that this research would help create awareness on medication reconciliation among pharmacists in developing countries, with a view to reducing medication-related patient harm.

## Background

Medication error is defined as any preventable event that may cause or lead to inappropriate medication use or patient harm while the medication is in the control of the health care professional, patient, or consumer [1]. Medication errors could serve as a source of economic burden to health services, with substantial negative health and economic consequences such as increased cost of treatment and mortality [2–5]. Some of the unwanted consequences of medication errors include adverse drug reactions, inadequate patient adherence and low quality of life [6]. Medication discrepancy, which is a type of medication error, could also arise during admission, transition, and discharge of patients from an institution [7, 8]. Several studies showed that discrepancies between medications prescribed and those taken by the patients may cause harm [1, 9, 10]. Such harms include hypoglycemia because of the administration of fast-acting insulin to a patient who was not on insulin and worsening of atrial fibrillation for a patient whose warfarin prescription was omitted.

Medication reconciliation, which is intended to minimize medication discrepancies and possible incidence of needless hospital readmissions,[11] is the comprehensive evaluation of a patient’s medication regimen any time there is a change in therapy in an effort to avoid medication errors such as omissions, duplications, dosing errors, or drug interactions, as well as to observe compliance and adherence patterns [12]. Medication reconciliation decreases incongruities between medications orders and the drugs taken by patients, when adequately implemented [13].

Medication reconciliation is an effective strategy to alleviate the danger and cost linked with medication errors during hospital admission and avoidable readmission [14]. A study carried out in the United States of America estimated 52% reduction of expected total cost of preventable adverse drug events from 472 US dollars for a patient receiving usual care to 266 US dollars for a patient receiving medication reconciliation intervention [15]. A potential net cost benefit of 103 euros per patient was reported by another study done in the Netherlands [16]. Another study carried out in the United Kingdom reported up to 80 pounds cost saving on preventable adverse drug events per patient for medication reconciliation carried out [17]. Several studies [15–18] and others have shown that pharmacists take more detailed medication history. The value of inclusion of pharmacists in medication reconciliation processes in acute care setting has been established by several studies [19, 20]. However, in Nigeria, publications on medication reconciliation are rare and anecdotal evidence show that medication reconciliation is not a structured practice, if done at all.

The purpose of this study was to evaluate background extent of medication reconciliation practice, and the effect of educational intervention on pharmacists’ practice of medication reconciliation among ambulatory diabetes and hypertensive patients in two tertiary hospitals in Nigeria.

## Methods

### Study design and setting

A mixed-method non-randomised clinical trial was carried out at two teaching healthcare facilities in Nigeria. The study was carried out at the University College Hospital, Ibadan (intervention site), a 950-bed teaching hospital affiliated with University of Ibadan. The University of Ilorin Teaching Hospital, Ilorin (control site) is a 650-bed teaching hospital affiliated with University of Ilorin. Both sites are major referral centers and centers of excellence for undergraduate and postgraduate training for physicians, pharmacists, nurses, and other healthcare practitioners in Nigeria. The study was carried out for a duration of 12 months.

### Inclusion and exclusion criteria

Pharmacists who gave their informed consent to participate in the study were recruited at both sites. Undergraduate pharmacy students on experiential rotation were excluded from the study. Patients (18 years and above) diagnosed with diabetes and/or hypertension who visited the Endocrinology or Cardiology Clinics were enrolled for the study. Patients who were not on medications for diabetes or hypertension, or those who did not consent to participate in the study were excluded.

### Data collection instruments

Three semi-structured questionnaires (Q1, Q2 and Q3) were used as the data collection instrument. The questionnaires were developed by the authors based on their teaching and practise experience, and extensive literature review [11–13, 17, 21–24]. The first questionnaire (Q1) which was a 22-item was interviewer-administered to patients by the principal investigator to indirectly assess pharmacists’ medication reconciliation practice. The Q1 comprised Section A, which contained items that addressed socio-demographic characteristics of the patients such as age, gender, education and occupation. Section B contained items which addressed patients’ medication reconciliation such as asking if they brought their medication pack from home to facilitate medication reconciliation process, medication history taking, including prescribed and over-the-counter medications, discontinued medications. Two questionnaires (Q2 and Q3) were used for pharmacists’ data collection. The second questionnaire (Q2) was an 18-item medication reconciliation intervention follow up form designed for data collection at one, three and six months post-general educational intervention. The questionnaire (Q2) was self-administered to the pharmacists to directly evaluate their medication reconciliation comprised Sections A and B. Section A contained the pharmacists’ socio-demographic information, and Section B contained questions on details of the medication reconciliation practice of the pharmacists, such as, frequency of practice, informing patients to come along with their medication packs for clinic visits, documentation of practice, identification and resolution of drug therapy problems. The third 8-item questionnaire (Q3) was designed to generate detailed information on pharmacists’ medication reconciliation practice post-focused educational intervention. Aside from socio-demographic characteristics, Q3 addressed details on patients’ previous and current medications, identification and resolution of medication discrepancies and drug therapy problems. The Q3 was interviewer-administered to patients by pharmacists recruited for the focused educational intervention to generate consistent data on their medication reconciliation practice.

The data collection instrument for patients was pretested for face validity among 34 diabetes and/or hypertensive patients at Catholic Hospital, Oluyoro, Ibadan. Also, pharmacists’ data collection instruments were pretested among 12 pharmacists at the University Health Services, University of Ibadan and Military Hospital, Ojoo, Ibadan. Content validity of all the questionnaires was done by three faculties at the Department of Clinical Pharmacy and Pharmacy Administration, Faculty of Pharmacy, University of Ibadan.

### Sample size determination

Sample size for the patient participants was determined using the following equation [25].

1 $$\mathrm{M }= 2{[{\mathrm{Z}}_{(1-\mathrm{\alpha }/2)}+{\mathrm{Z}}_{(1-\upbeta )}]}^{2/\updelta 2}$$

$$\mathrm{\alpha }= 5\mathrm{\% }(0.05),\upbeta = 20\mathrm{\% }(0.2),\updelta = 50\mathrm{\% }(0.5)$$

Equal distribution of participants to each of the treatment groups was done. Two-sided statistical tests were carried out, assuming a normal distribution. To identify effect of treatment with 80 percent power at 5% significance level, the emblematic standard values were used at:

$$\mathrm{Significance level}, {\mathrm{Z}}_{(1-\mathrm{\alpha }/2)}\mathrm{ at }5\mathrm{\% }= 1.96$$

$$\mathrm{Power}, {\mathrm{Z}}_{(1-\upbeta )}\mathrm{ at }80\mathrm{\% }= 0.8416$$

${\mathrm{Z}}_{(1-\mathrm{\alpha }/2)}\mathrm{ and }{\mathrm{Z}}_{(1-\upbeta )}$= Normal distribution % points for significance level and power, respectively

δ = standardized difference (i.e., treatment difference)

From equation (1) above

$$2{[1.96 + 0.8416]}^{2}/0.52= 15.6979/0.25 = 62.8$$

Considering 10% attrition, the sample size was determined to be 70 patients per group. The calculated sample size was used as a guide to recruit participants and total sampling was adopted for recruiting the pharmacists.

### Recruitment of participants and data collection

Sequel to acquiring ethics approval from each hospital review board, the approvals of heads of different units/departments where the study was undertaken were also secured. Total sampling of the entire pharmacists at the control and intervention sites was adopted for the study. The purpose of the study was explained to all the pharmacists and consulting physicians in each hospital. Thereafter, pharmacists were visited in their different units and the questionnaire administered to those who gave informed consent. The study, which was a mixed-method non-randomised clinical trial utilised self-administered questionnaire (Q2) among 146 pharmacists (intervention site-85; control site-61) to directly evaluate their medication reconciliation practice. Ambulatory diabetes and/or hypertensive patients were visited on their respective clinic days during which the purpose of the study was explained in English and Yoruba (local language), as required. Interviewer-administered semi-structured questionnaires (Q1 and Q3) were employed to carry out medication reconciliation for a total of 660 ambulatory patients (Q1 for 520 and Q3 for 140 diabetes and/or hypertension patients) throughout the study in different cohorts to indirectly evaluate the pharmacists’ medication reconciliation practice, at both sites. Patients with diabetes and/or hypertension were targeted because of the prevalence of the two diseases in Nigeria (5.77% for diabetes and 30.6% for hypertension) [26, 27]. They were considered high-risk patients for medication reconciliation due to chronic medication administration, as well as the possibility of presence of other comorbidities.

Baseline medication reconciliation practice of the pharmacists was indirectly evaluated as the principal investigator carried out medication reconciliation among a cohort of 334 (intervention-183; control-151) out of the 660 patients. Thereafter, a general educational intervention was carried out among the 85 pharmacists, hereafter referred to as intervention pharmacists, in the intervention group to address the medication reconciliation practice gaps observed at baseline. The semi-structured questionnaire (Q2) was administered to pharmacists at the intervention site at one, three and six months to assess their medication reconciliation practice of the pharmacists after the general educational intervention. The effect of the intervention on pharmacists’ medication reconciliation practice was also indirectly evaluated using Q1 as the principal investigator carried out medication reconciliation among cohorts of 96 (intervention-46; control-50) patients at three months and 90 (intervention-44; control-46) at six months postintervention. This was followed by a focused educational intervention for 15 pharmacists (a subset of the initial 85 intervention pharmacists) at the Geriatric Center of the intervention site. The data collected by the 15 pharmacists after carrying out medication reconciliation for a cohort of 140 patients was independently reviewed by three experts, who were faculties at the Department of Clinical Pharmacy and Pharmacy Administration of the University of Ibadan. The three experts in this study were faculties at the Department of Clinical Pharmacy and Pharmacy Administration, Faculty of Pharmacy, University of Ibadan, Nigeria. They were selected based on their competence in the core areas of medication reconciliation, which includes comprehensive medication history taking, documentation of clinical care activities, identification and resolution of drug therapy problems as well as medication discrepancies. The criteria to define them as experts in clinical pharmacy includes the fact that they have several years of teaching and research experience in Clinical Pharmacy. The three experts comprised an Associate Professor and two Senior Lecturers who are well versed in intervention studies aimed at improving the quality of care provided by pharmacists for patients. They have also made extensive contributions in Clinical Pharmacy with several articles published in both local and international peer-reviewed journals. Outcomes measured included identification and resolution of drug therapy problems, medication discrepancies and patients who brought their medication packs for clinic appointment.

### Educational interventions

Two educational interventions were carried out by the principal investigator during the study, who is a faculty and a doctorate student working on medication reconciliation at the Department of Clinical Pharmacy and Pharmacy Administration, Faculty of Pharmacy, University of Ibadan, Nigeria. He underwent a two-week training on “Best Clinical Practices” organized by University of Nigeria Teaching Hospital (UNTH) in collaboration with the Nigerian Association of Pharmacists and Pharmaceutical Scientists in the Americas (NAPPSA) at UNTH, Nigeria in 2015. He also had a 6-week International Pharmacists’ Enrichment Programme at Howard University, Washington DC, with focus on medication reconciliation, jointly organized by FIP-Pharmabridge and Howard University in 2016.

The first intervention was a general intervention carried out among the entire 85 pharmacists recruited for the study at the intervention site. This intervention was aimed at educating the pharmacists on comprehensive medication history taking, effective communication with patients and other healthcare team members, identification and resolution of drug therapy problems, and the concept and practice of medication reconciliation. The intervention, which lasted for four hours, comprised didactic lectures, role-plays, and case-reviews on skills required for medication reconciliation. The second intervention was a focused intervention which involved a detailed follow up educational intervention with emphasis on consistent documentation of medication reconciliation practice. The intervention, which lasted for one hour, consisted of hands-on practice on medication reconciliation, documentation of clinical practices, detection and resolution of drug therapy problems and medication discrepancies. The questionnaire (Q3) for consistent medication reconciliation data collection designed for this phase was utilized by the 15 pharmacists for data collection. Both educational interventions were carried out at the Pharmacy Department of the intervention site.

### Data analysis

Data was summarized with descriptive and inferential statistics using SPSS for Windows Version 23.0 (IBM Corp, New York, USA). Normal distribution of the data was evaluated using Kolmogorov-Smirnov test. Inferential statistics such as Fisher’s exact test was done to compare associations between absence/presence of medication discrepancy among patients at the intervention and control sites. Pearson product-moment correlations analysis was carried out to investigate relationships between patients’ medication discrepancy and comorbidity, number of medication(s) and educational level. Independent-samples t-test was used to evaluate the difference between average medication discrepancy among patients at the intervention and control sites. One-way analysis of variance compared patients’ medication discrepancies at the intervention and control sites over the study period. The level of significance was set at *p* < 0.05. Fleiss’ Kappa inter rater analysis was employed to find out the level of agreement in the medication reconciliation practice assessment done by the three experts.

## Results

**Fig. 1:**
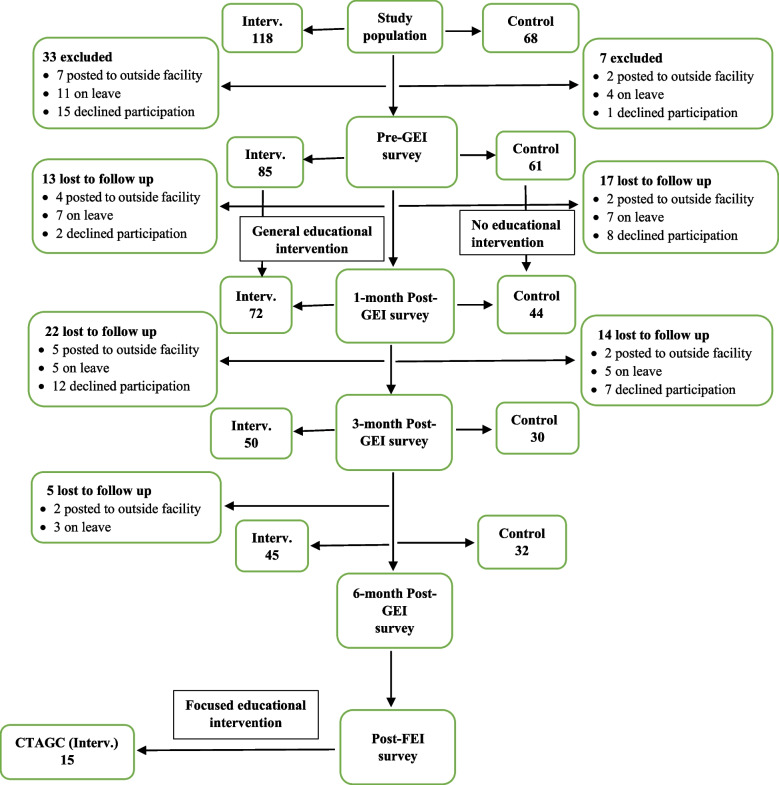
CONSORT for study pharmacist-participants. Interv. = Intervention PI = Postintervention CTAGC = Chief Toni Anenih Geriatric Center. GEI = General educational intervention FEI = Focused educational intervention

**Fig. 2:**
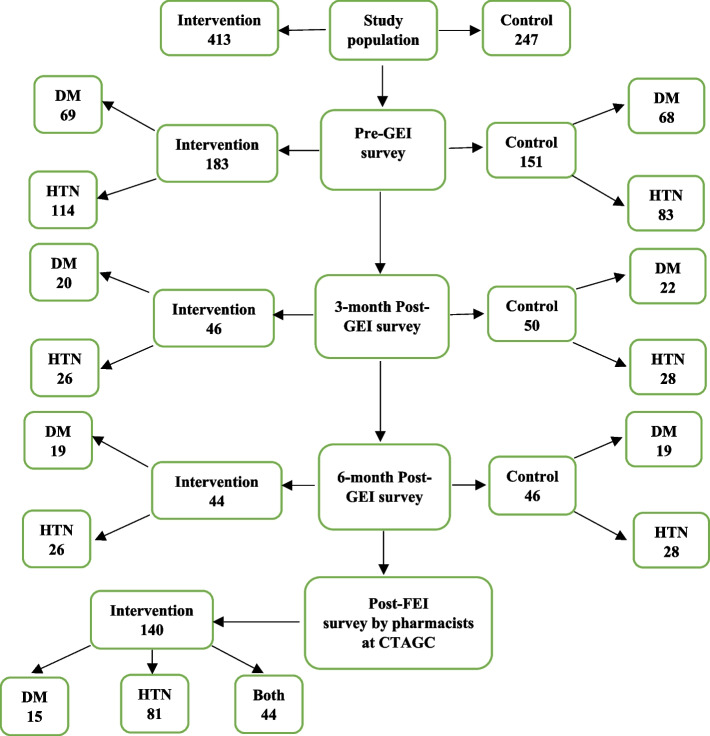
CONSORT for study patient-participants. CTAGC = Chief Toni Anenih Geriatric Center Both = Diabetes and Hypertension. CONSORT = Consolidated Standards of Reporting Trial PI = Postintervention. GEI = General educational intervention FEI = Focused educational intervention. NB: Patients selected for each phase of the study were cohorts and not the same sets. Total patient population = 660

Out of the 146 pharmacists recruited, 35 (35.0%) and 14 (23.0%) at the intervention and control sites, respectively, had additional qualifications aside from the Bachelor of Pharmacy degree. At the intervention site, the average years of work experience as hospital pharmacist was 7.76 ± 8.15 while at the control site it was 7.23 ± 9.23. Other demographic characteristics of the pharmacists are as shown in Table 1. Forty pharmacists (49.4%) were lost to follow-up at the intervention site and 31 pharmacists at the control site. The Consolidated Standards of Reporting Trials (CONSORT) for the pharmacist-participants and patients in the study are as shown in Figures 1 and 2, respectively. There were 115 (62.8%) and 70 (46.4%) female patients recruited at the intervention and control sites, respectively at baseline. Detailed sociodemographic characteristics of the patients is presented in Table 2.

**Table 1:** Demographic characteristics of pharmacists

**Variables**	**Intervention (*n* = 85)**		**Control (*n* = 61)**	
	**Frequency**	**Percent**	**Frequency**	**Percent**
**Gender**				
Female	57	67.1	30	49.2
Male	28	32.9	31	50.8
**Hospital cadre**				
Intern Pharmacist	27	31.8	28	45.9
National Youth Service Scheme Pharmacist	0	0	1	1.6
Pharmacist I	24	28.2	11	18.0
Senior Pharmacist	NA	NA	2	3.3
Principal Pharmacist	2	2.4	6	9.8
Chief Pharmacist	13	15.3	4	6.6
Assistant Director of Pharmaceutical Services	11	12.9	0	0
Deputy Director of Pharmaceutical Services	7	8.2	9	14.8
Director of Pharmaceutical Services	1	1.2	0	0
**Years of hospital pharmacy experience**				
1 – 5	45	52.9	42	68.9
6 – 10	16	18.8	4	6.6
> 10	24	28.2	15	24.6
**Educational qualification(s)**				
B. Pharm only	48	56.5	46	75.4
FPCPharm	13	15.3	6	9.8
MBA	0	0	1	1.6
PhD	1	1.2	0	0
MBA + FPCPharm	1	1.2	1	1.6
M. Pharm/M. Sc./MPH	9	10.6	4	6.6
M. Sc. + FPCPharm	13	15.3	3	4.9

*B. Pharm* Bachelor of Pharmacy, *MBA* Master of Business Administration, *M. Sc.* Master of Science, *MPH* Master of Public Health, *M. Pharm* Master of Pharmacy, *PhD* Doctor of Philosophy, *FPCPharm* Fellow, Postgraduate College of Pharmacists, *NA* Designation of Senior Pharmacists is not used at the intervention site

**Table 2:** Demographic characteristics of patients

**Variables**		**Baseline**		**3-month post-GEI**		**6-month post-GEI**	
		**Intervention (*n*=183)**	**Control (*n*=151)**	**Intervention (*n*=46)**	**Control (*n*=50)**	**Intervention (*n*=44)**	**Control (*n*=46)**
		**Frequency (%)**	**Frequency (%)**	**Frequency (%)**	**Frequency (%)**	**Frequency (%)**	**Frequency (%)**
**Gender**	Female	115 (62.8)	70 (46.4)	21 (45.7)	25 (50.0)	29 (65.9)	25 (54.3)
	Male	68 (37.2)	81 (53.6)	25 (54.3)	25 (50.0)	15(34.1)	21 (45.7)
**Male: Female ratio**		1: 1.7	1: 0.9	1: 0.4	1: 1	1: 1.9	1: 1.2
**Age category (years)**	< 50	30 (16.4)	27 (17.9)	4 (8.7)	24 (48.0)	0 (0.0)	7 (15.2)
	50 – 59	14 (7.7)	41 (27.2)	9 (19.6)	13 (26.0)	0 (0.0)	13 (28.3)
	60 – 69	68 (37.2)	43 (28.5)	17 (37.0)	8 (16.0)	19 (43.2)	9 (19.6)
	> 69	71 (38.8)	40 (26.5)	16 (34.8)	5 (10.0)	25 (56.8)	17 (37.0)
**Religion**	Christianity	141 (77.0)	64 (42.4)	30 (65.2)	41 (82.0)	37 (84.1)	35 (76.1)
	Islam	42 (23.0)	87 (57.6)	16 (34.8)	9 (18.0)	7 (15.9)	11 (23.9)
**Level of education**	None	19 (10.4)	22 (14.6)	8 (17.4)	0 (0.0)	5 (11.4)	0 (0.0)
	Primary	36 (19.7)	35 (23.2)	9 (19.6)	5 (10.0)	11 (25.0)	3 (6.5)
	Secondary	48 (26.2)	36 (23.8)	8 (17.04)	3 (3.0)	12 (27.3)	16 (34.8)
	Tertiary	80 (43.7)	58 (38.4)	21 (45.7)	42 (84.0)	16 (36.4)	27 (58.7)

*Post-GEI* Post-general educational intervention

Data analysed was normally distributed. The number of patients with medication discrepancy (intervention site vs control site) was 80 (43.7%) vs 54 (35.8%) (*p* = 0.086) at baseline, 20 (43.5%) vs 30 (60.0%) (*p* = 0.078) at three months postintervention, and 11 (25.0%) vs 30 (65.2%) (*p* < 0.001) at six months postintervention. Average number of medication discrepancy observed among the cohorts of patients at the intervention site was 0.76 ± 0.68, 0.57 ± 0.75 and 0.43 ± 0.66 at baseline, three-, and six-months postintervention, respectively. A statistically significant difference was only observed between baseline and 6-month post-intervention (*p* = 0.013). At the control site, the average number of medication discrepancy observed was 0.74 ± 0.84, 0.72 ± 0.95 and 0.67 ± 0.76 at baseline, three- and six-months postintervention, respectively. No significant difference was observed in the average number of medication discrepancy. Medication discrepancy had a significant correlation with number of medications used (*r* = 0.212, *p* < 0.001) and comorbidity (*r* = 0.135, *p* < 0.001), but no significant correlation with educational level (*r* = -0.091, *p* = 0.095), gender (*r* = -0.034, *p* = 0.533) or age (*r* = 0.062, *p* = 0.251) of the patients.

Thirty-five, 66 and 48 drug therapy problems were detected by 31 (43.1%), 33 (66.0%) and 32 (71.1%) intervention pharmacists at 1-, 3- and 6-month post-general educational intervention, respectively. The general educational intervention led to an increase in the number of pharmacists who reported documenting their care activities from 33.3% at 1-month to 64.4% at 6-month post-intervention. Likewise, pharmacists who reported informing patients to bring their medication packs along for hospital appointments increased from 43.1% (one month) to 75.6% (six months) after the intervention.

The potential clinical implications of medication discrepancies among the 520 patients who participated at different periods from baseline to 6-month post-general educational intervention is as shown in Table 3. At baseline, 48.6% vs 57.0% of patients (intervention site vs control site) brought their medication packs along for clinic appointment (*p* = 0.152). There was an increase at the intervention site at 3-month postintervention (intervention site - 71.7%; control site - 44.0%) (*p* = 0.008) and at 6-month postintervention (intervention site - 75.0%; control site - 43.5%) (*p* = 0.003).

**Table 3:** Potential clinical implications of medication discrepancy detected and resolved from baseline to 6-month post-GEI by the principal investigator

**Discrepancies identified**	**Potential clinical implications of discrepancy**	**Description of medication discrepancy**	**Baseline**			**3 months**			**6 months**		
			**Interv. (*n*=183)**	**Control (*n*=151)**	***p* value**^**a**^	**Interv. (*n*=46)**	**Control (*n*=50)**	***p* value**^**a**^	**Interv. (*n*=44)**	**Control (*n*=46)**	***p* value**^**a**^
			**Frequency (%)**			**Frequency (%)**			**Frequency (%)**		
**Total number of medication discrepancies**			139	112		26	36		14	31	
Omission of medication	Therapeutic failure	Omission of medication for diabetes	13 (9.4)	3 (2.7)	0.001*	2 (7.7)	2 (5.6)	1.000	0(0.0)	1 (3.2)	0.617
		Omission of antihypertensives	11 (7.9)	1 (0.9)		0(0.0)	0(0.0)		1 (7.1)	2 (3.6)	
	Suboptimal medication therapy	Omission of key medication	18 (12.9)	14 (12.5)	1.000	3 (11.5)	6 (16.7)	0.490	2 (14.3)	7 (22.6)	0.158
	Poor quality of care	Medication discontinued by patient	23 (16.5)	14 (12.5)	0.384	2 (7.7)	5 (13.9)	0.438	1 (7.1)	4 (12.9)	0.361
	Possible increased risk of cardiovascular disease	Omission of Aspirin/Clopidogrel	14 (7.0)	7 (3.9)	0.028*	1 (1.7)	2 (3.3)	0.618	0 (0.0)	2 (3.6)	0.242
		Omission of statins	10 (5.0)	2 (1.1)		0 (0.0)	1 (1.6)		0 (0.0)	1 (1.8)	
Different dose/frequency of medication	Therapeutic failure	Too low dose of medication(s)	8 (5.8)	16 (14.2)	0.034*	2 (7.7)	2 (5.6)	1.000	0 (0.0)	0 (0.0)	c
	Adverse drug reaction	Dose-related adverse drug reactions	5 (3.6)	9 (8.0)	0.174	3 (11.5)	2 (5.6)	0.668	1 (7.1)	1 (3.2)	1.000
	Toxicity	High dose of digoxin	3 (2.2)	4 (3.6)	0.706	1 (3.8)	0 (0.0)	0.479	0 (0.0)	0 (0.0)	c
	Increased medication cost	Higher dose related cost increment	11 (8.0)	24 (21.5)	0.004*	4 (15.4)	3 (8.4)	0.242	4 (28.5)	5 (16.1)	0.412
Duration of medication exceeded	Adverse drug reaction	Prolonged use of NSAID	1 (0.7)	1 (0.9)	1.000	0 (0.0)	1 (2.8)	1.000	0 (0.0)	0 (0.0)	c
	Safety	Proton pump inhibitor prolonged use	2 (1.4)	1 (0.9)	1.000	3 (11.5)	0 (0.0)	0.106	1 (7.1)	0 (0.0)	0.489
Substitution of prescribed medication by patients	Adverse drug reaction	Aspirin substituted for Clopidogrel	2 (1.4)	0 (0.0)	0.503	0 (0.0)	1 (2.8)	1.000	0 (0.0)	2 (6.4)	0.495
	Therapeutic failure	Biguanide substituted for insulin	1 (0.7)	1 (0.9)	1.000	0 (0.0)	1 (2.8)	1.000	0 (0.0)	0 (0.0)	c
	Ineffective therapy	ACEIs substituted for CCBs	0 (0.0)	0 (0.0)	c	0 (0.0)	2 (5.6)	0.496	1 (7.1)	0 (0.0)	0.489
Duplication of medication with different brands	Adverse drug reaction	Took 2 different brands of Metformin	0 (0.0)	1 (0.9)	0.452	0 (0.0)	0 (0.0)	c	1 (7.1)	0 (0.0)	c
	Toxicity	Took 2 brands of digoxin	4 (2.9)	2 (1.8)	0.693	1 (3.8)	1 (2.8)	1.000	0 (0.0)	0 (0.0)	c
	Increased medication cost	Higher cost for duplicated drugs	0 (0.0)	0 (0.0)	c	0 (0.0)	0 (0.0)	c	1 (7.1)	0 (0.0)	0.489
Additional medication not prescribed by physician	Dependence	Use of Bromazepam	0 (0.0)	1 (0.9)	0.452	1 (3.8)	0 (0.0)	0.479	1 (7.1)	0 (0.0)	0.489
	Increased medication cost	High cost of added medications	6 (4.3)	6 (5.4)	0.775	3 (11.5)	3 (8.3)	1.000	0 (0.0)	4 (12.9)	0.117
	Toxicity	Digoxin intake	3 (2.2)	1 (0.9)	0.630	0 (0.0)	1 (2.8)	1.000	0 (0.0)	1 (3.2)	1.000
	Adverse drug reaction	Took Nifedipine with Amlodipine	3 (2.2)	4 (3.6)	0.706	0 (0.0)	2 (5.6)	0.496	0 (0.0)	1 (3.2)	1.000
	Tolerance	Chronic use of salbutamol	1 (0.7)	0 (0.0)	1.000	0 (0.0)	1 (2.8)	1.000	0 (0.0)	0 (0.0)	c

*Interv* Intervention, *GEI* General educational intervention, *NSAIDs* Nonsteroidal Anti-Inflammatory Agents, *c* No statistics computed because the values are constant, *ACEIs* Angiotensin Converting Enzyme Inhibitors

^a^Test statistics = Chi square (Linear-by-linear association), *CCBs* Calcium Channel Blockers

^*^ *p* < 0.05

Fifteen pharmacists participated in medication reconciliation carried out among a cohort of 140 patients at the Geriatric Center. There were 78 (55.7%) female patients. Fifteen (5.4%) patients had diabetes, 81 (28.9%) hypertension and 44 (15.7%) had both diabetes and hypertension. The average medications prescribed per patient was 4.96 ± 1.94. One hundred and fifteen (82.1%) patients brought their medication packs along for their hospital appointment. The possible consequences of the medication discrepancies detected and resolved by the intervention pharmacists is shown in Table 4. Out of a total of 695 medications taken by the patients, 75 (10.8%) medication discrepancies were detected among 42 (30%) patients. There were 35 (46.7%) unprescribed/self-medications, 21 (28.0) medication duplications, 6 (8.0%) different dose/frequency of administrations, 5 (6.7%) wrong durations, 6 (8.0%) substitutions, and 2 (2.7%) omissions.

**Table 4:** Potential clinical implications of medication discrepancies detected and resolved by the 15 intervention pharmacists at the geriatric center

**Discrepancies identified**	**Description of medication discrepancy**	**Potential clinical implications of discrepancy**	**Step taken by pharmacist**	**Physician’s response to the query raised**	**n (%)**	**% Accuracy**
**Unprescribed medications/Self-medication**	Took Tab Prednisolone recommended by a friend for pain	Worsening of disease condition	Patients were advised to discontinue the specific unprescribed medications due to potential adverse events.	Not required	1 (1.3)	
	Antibiotics	Disruption of normal microbial flora		Not required	11 (14.7)	
	Tab Ketoconazole	Possible liver impairment, stomach pain, skin rash, orthostatic hypotension		Not required	1 (1.3)	
	Tab Potassium	Risk for cardiac arrhythmias		Not required	1 (1.3)	
	Cap Omeprazole	Risk for pneumonia		Not required	1 (1.3)	
	Antihypertensive medications	Risk for hypotension		Not required	4 (5.3)	
	NSAIDs and other pain relief medications	Risk for end organ impairment especially the kidneys		Not required	13 (17.3)	
	Anxiolytics	Increased risk for falls		Not required	1 (1.3)	
	Took opioid pain relief medications	Dependence		Not required	2 (2.7)	100%
	**Total**				**35 (46.7)**	
**Duplication**	Two different Atorvastatin taken together	Increased risk for adverse medication reactions Risk for end organ impairment	Patients were advised to discontinue the duplicate medication due to potential adverse events. Referred to physician	Not required	3 (4.0)	
	Two brands of Cap Doxycycline taken together			Not required	1 (1.3)	
	Two NSAIDs taken together			Not required	2 (2.7)	
	Two brands of Cap Omeprazole taken together			Not required	1 (1.3)	
	Duplicates of antihypertensive medications	Hypotension risk		Not required	8 (10.7)	
	Duplicates of Tab Metformin taken together	Hypoglycemia risk		Not required	3 (4.0)	
	Aspirin and Clopidogrel taken together	Risk for bleeding		Not required	2 (2.7)	
	Tab Potassium and Tab Spironolactone taken together	Risk for cardiac arrhythmias		Tab Potassium withdrawn	1 (1.3)	80.8%
	**Total**				**21 (28.0)**	
**Different dose/ frequency of administration**	Tab Nifedipine 30mg bd vs daily	Hypotension risk	Patients were asked to take appropriate doses	Not required	1 (1.3)	
	Higher dose of statins taken by patient	Risk for adverse effects		Not required	2 (2.7)	
	Higher dose of diabetes medications taken	Hypoglycemia risk		Not required	2 (2.7)	
	Tab Metformin 500mg bd vs tds	Suboptimal dose		Not required	1 (1.3)	100%
	**Total**			Not required	**6 (8.0)**	
**Duration**	Patient taking discontinued antibiotics	Disruption of normal microbial flora	Medications withdrawn	Not required	2 (2.7)	
	Patient taking discontinued PPIs	Risk for pneumonia			3 (4.0)	83.3%
	**Total**				**5 (6.7)**	
**Substitution**	Tab Aspirin vs Tab Clopidogrel	Gastric ulceration risk	Referred to physician	Tab Clopidogrel retained and Aspirin discontinued	1 (1.3)	
	Tab S-Amlodipine 5mg vs Tab Amlodipine 5mg	Hypotension	Referred to physician	Tab Amlodipine 5mg retained	1 (1.3)	
	Herbal remedies substituted for prescribed OHAs	Poorly controlled diabetes	Patient advised to reinstitute OHAs	Not required	1 (1.3)	
	Herbal remedies vs Antihypertensive medications	Poorly controlled hypertension		Not required	1 (1.3)	
	Tab Bisoprolol + Tab Ramipril substituted by patient for Tab Uperio^®^	Worsening heart failure	Referred to physician	Patient counseled on adherence; Bisoprolol + Ramipril discontinued	1 (1.3)	
	Tab Simvastatin 40mg vs Tab Atorvastatin 40mg	Suboptimal dose	Referred to physician	Tab Atorvastatin retained, Simvastatin discontinued	1 (1.3)	85.7%
	**Total**				**6 (8.0)**	
**Omission**	Tab Atorvastatin	Increase risk for CVD	Referred to physician	Tab Atorvastatin prescribed	1 (1.3)	
	Arthritis medication	Poor quality of patient’s life	Referred to physician	Diclofenac prescribed	1 (1.3)	40%
	**Total**				**2 (2.7)**	

% accuracy = # Medication discrepancies detected by pharmacists/# Medication discrepancies detected by the Experts x 100%

*NSAIDS* Non-Steroidal Anti-inflammatory Agents, *OHAs* Oral hypoglycemic agents, *PPIs* Proton pump inhibitors, *Uperio*^*®*^ Sacubitril + Valsartan, *CVD* Cardiovascular disease

The inter-rater reliability analysis showed very good agreement, K = 0.990 (95% CI, 0.986 to 0.992), *p* < 0.001. Based on the review done by the experts, the pharmacists missed five cases of medication duplication, one case each of wrong duration and medication substitution, and three cases of omission giving a percentage accuracy of 80.8%, 83.3%, 85.75 and 40%, respectively. The pharmacists had 100% accuracy with detecting unprescribed medication(s) and wrong medication doses.

## Discussion

This study revealed poor baseline medication reconciliation practice among pharmacists at both study sites. The focused educational intervention especially improved the practice of medication reconciliation by pharmacists at the intervention site. There was a reduction in medication discrepancies and an increase in detection and resolution of drug therapy problems by the intervention pharmacists.

As evidenced by the lack of documented cases on medication reconciliation at baseline, and the slow build up to the adoption of the process during the study, the practice of medication reconciliation was a non-deliberate, haphazard, and unmonitored practice which was seldom done by a few pharmacists at both sites. This might be because medication reconciliation is not yet an established component of pharmacy practice in Nigeria [28]. Also, there is no regulatory policy insisting on medication reconciliation as one of the accreditation criteria for Nigerian hospitals, as operational in many developed countries [29, 30]. Documentation of clinical care activities was also found not to be common in Nigeria. Previous studies in southwest Nigeria showed that pharmacists tend not to document their clinical practices. Aje and Erhun (2016) [31], and Aje and Davies (2016) [32] showed that less than half of community pharmacists who made interventions on point-of-care test results and detected drug therapy problems did not document these activities. Suleiman and Onaneye (2011) showed that even though over half of the community and hospital pharmacists detected mistakes in patients’ prescriptions in a particular study, none of the mistakes and corresponding interventions were documented [33]. A similar study in southeast Nigeria by Offu (2019) showed that about one-quarter of community pharmacists document their care activities [34]. Another study carried out in southeast Nigeria, using self-report among pharmacists in two tertiary hospitals, showed that over one-tenth of the pharmacists were not documenting their care activities [35]. Personal communications with some hospital pharmacists showed that the documentation of pharmacists’ clinical practices is not allowed in patients’ case notes where it could be accessed by other healthcare professionals. The importance of documenting clinical care activities has been demonstrated by many studies [35, 36]. Cipolle et al described the process of documentation of care activities as being vital as it is the only proof of work done, while enhancing patient follow up [37]. Also, Zierler-Brown et al asserted that documentation of pharmacists’ care activities serves as a template to show quality of service, proof of pharmacists’ role in patient care, quality assurance device for standards of medical practice and eventually enhancing team building among healthcare practitioners [38]. A well carried out medication reconciliation is expected to result in a comprehensive medication list for both the patient and other healthcare professionals for seamless continuity of medical care [13].

One of the processes during medication reconciliation is the review of patients’ past medications and comparing it with the newly prescribed medications. Healthcare practitioners, especially pharmacists, need to regularly educate their patients on the need to always bring their medications along for hospital visits. Studies from developed countries show that patients bringing their medication packs along for hospital appointments vary from one place to another [19, 39–41]. The act of bringing medication packs aid in spotting potential medication interaction, omission, and/or duplication of medication. This could help in two ways, *viz*, evaluation of patient medication adherence and medication reconciliation. Though bringing the medication pack along for pill count is not a perfect method for evaluating medication adherence, it can be used in addition to other methods to get a clearer picture of adherence/nonadherence. Aside from providing the opportunity for indirect measurement of medication adherence by pill count, taking medication packs along for hospital appointments make it easier to obtain an accurate list of medications being taken by the patient. When not brought along, recall bias by the patients could be a major challenge in the process of medication reconciliation. In this study, presence of medication packs was mainly used to obtain an accurate list of medications being taken by the patient.

Medication reconciliation is a tool for detecting discrepancies in prescribed medications in diverse healthcare settings, or at different levels of care to update patient’s medication and avoid medication errors [21]. The indirect measurement of medication discrepancy in this study at baseline showed no significant difference at both sites. However, the difference between medication discrepancies observed during the study was not significant until six months post-general intervention, indicating a slow build up in the practice of medication reconciliation among the pharmacists. The low level of pharmacist-patient ratio could be a factor, indicating that more time may be required to observe positive changes in medication reconciliation practices sequel to an educational intervention. The pharmacists who underwent focused educational intervention were able to do proper documentation and prevent medication-related patient harm during the medication reconciliation process. Ability of the pharmacists to reconcile medications, identify and resolve adverse effects of drugs may have had a potential impact in preventing patient-related harms in this study.

A significant reduction in the occurrence of medication discrepancy from 43.75% to 25.0% at the intervention site, after the general educational intervention. Medication discrepancy observed among patients in related studies showed a range of 33.2% - 86.1% with a range of six to ten medications taken by the patients [19, 39, 22-24,]. It is expected that a much lower occurrence of medication discrepancy would be reported in developed countries where medication reconciliation is already an institutionalized practice. However, in the present study with a range of two to ten medications, the occurrence of medication discrepancies was significantly reduced from 43.75% to 25.0%. The higher average number of medications taken by patients in developed countries could be responsible for the higher medication discrepancies. This is logical, since medication discrepancy is likely to increase with increasing number of medications, especially as found in a geriatric population [42].

Since patient safety is the primary goal of medication reconciliation, this study equipped pharmacists in the intervention site with knowledge and skill to improve patient safety by reducing medication discrepancies. Therefore, an institutionalized medication reconciliation practice will help to eventually reduce medication discrepancy in the long run.

### Study limitations

The attrition rate observed among pharmacists at both sites was quite high. This level of attrition was because a few pharmacists were posted out of the study sites, some dropped out for personal reasons, while some were on leave at different times during the study period. Since the study was carried out among ambulatory diabetes and hypertensive patients alone, the results may not be generalizable to diabetes and/or hypertensive patients in other transitions of care. In the practice of medication reconciliation, self-report was used by the pharmacists. This method of data collection could be susceptible to bias [43]. Non-participatory observation by the principal investigator while medication reconciliation was being practised by the pharmacists could have been a better method. But this could also have introduced a Hawthorne effect on the pharmacists. Hawthorne effect can also not be ruled out from the repeated use of the same questionnaire among the pharmacists, post-general intervention.

## Conclusion

The educational interventions improved intervention pharmacists’ medication reconciliation practice and led to prevention of medication-related harm to patients. It is recommended that this intervention be replicated in more hospitals in Nigeria to encourage implementation of best practices.

## Data Availability

The datasets used and/or analysed during the current study available from the corresponding author on reasonable request.

## Abbreviations

ACEIs: Angiotensin Converting Enzyme Inhibitors
B. Pharm: Bachelor of Pharmacy
CCBs: Calcium Channel Blockers
CONSORT: The Consolidated Standards of Reporting Trials
CVD: Cardiovascular disease
FPCPharm: Fellow, Postgraduate College of Pharmacists
GEI: General educational intervention
Interv.: Intervention
M. Pharm: Master of Pharmacy
M. Sc.: Master of Science
MBA: Master of Business Administration
MPH: Master of Public Health
NSAIDs: Nonsteroidal Anti-Inflammatory Agents
OHAs: Oral hypoglycemic agents
PhD: Doctor of Philosophy
Post-GEI: Post-general educational intervention
PPIs: Proton pump inhibitors
